# Time-Series Change Detection Using KOMPSAT-5 Data with Statistical Homogeneous Pixel Selection Algorithm

**DOI:** 10.3390/s25020583

**Published:** 2025-01-20

**Authors:** Mirza Muhammad Waqar, Heein Yang, Rahmi Sukmawati, Sung-Ho Chae, Kwan-Young Oh

**Affiliations:** 1 Satellite Image Application Team, CONTEC, Daejeon 34074, Republic of Korea; heein.yang@contec.kr (H.Y.); rahmi.sukmawati@contec.kr (R.S.); 2 Satellite Application Division, Korea Aerospace Research Institute (KARI), Daejeon 34133, Republic of Korea; shchae90@kari.re.kr (S.-H.C.); ohky@kari.re.kr (K.-Y.O.)

**Keywords:** change detection, statistical homogeneous pixels (SHP), KOMPSAT-5 amplitude change detection

## Abstract

For change detection in synthetic aperture radar (SAR) imagery, amplitude change detection (ACD) and coherent change detection (CCD) are widely employed. However, time-series SAR data often contain noise and variability introduced by system and environmental factors, requiring mitigation. Additionally, the stability of SAR signals is preserved when calibration accounts for temporal and environmental variations. Although ACD and CCD techniques can detect changes, spatial variability outside the primary target area introduces complexity into the analysis. This study presents a robust change detection methodology designed to identify urban changes using KOMPSAT-5 time-series data. A comprehensive preprocessing framework—including coregistration, radiometric terrain correction, normalization, and speckle filtering—was implemented to ensure data consistency and accuracy. Statistical homogeneous pixels (SHPs) were extracted to identify stable targets, and coherence-based analysis was employed to quantify temporal decorrelation and detect changes. Adaptive thresholding and morphological operations refined the detected changes, while small-segment removal mitigated noise effects. Experimental results demonstrated high reliability, with an overall accuracy of 92%, validated using confusion matrix analysis. The methodology effectively identified urban changes, highlighting the potential of KOMPSAT-5 data for post-disaster monitoring and urban change detection. Future improvements are suggested, focusing on the stability of InSAR orbits to further enhance detection precision. The findings underscore the potential for broader applications of the developed SAR time-series change detection technology, promoting increased utilization of KOMPSAT SAR data for both domestic and international research and monitoring initiatives.

## 1. Background

Change detection is a methodical process that examines a pair of images captured from the same scene at different times to identify and quantify changes that have occurred between the respective acquisition dates [1]. Synthetic aperture radar (SAR) change detection techniques have gained increasing importance due to SAR’s unique capabilities, such as its all-weather and day-and-night imaging capabilities, and its ability to penetrate through vegetation and measure subtle ground displacements. Key areas of focus in SAR-based change detection research include land cover change, urban expansion, natural disaster monitoring, and ground deformation analysis. SAR-based change detection has proven invaluable in assessing and managing natural disasters like earthquakes, landslides, and floods. For instance, InSAR techniques have been widely applied to measure surface deformations caused by seismic activities, allowing researchers to assess earthquake damage and tectonic activity with high spatial accuracy. Following major flood events, SAR intensity data enable rapid flood extent mapping due to SAR’s ability to penetrate cloud cover, which is critical for timely disaster response. Research also emphasizes landslide detection through SAR coherence loss mapping and multi-temporal analysis to identify areas with increased landslide risk, especially in regions with rugged topography.

Although SAR change detection has achieved significant advances, challenges remain in the form of high computational costs and limitations in accurately interpreting complex environments. Recent research has focused on overcoming these limitations through advanced pre-processing techniques, enhanced image segmentation, and deep learning methods. Speckle noise reduction methods, including non-local means filters and adaptive SAR despeckling techniques, are actively being studied to improve data quality. The fusion of SAR data with optical imagery and other remote sensing data sources is another growing trend, offering complementary information that enhances the reliability of change detection results. Additionally, multi-platform SAR systems, incorporating data from different satellites or different SAR sensors, are being explored to improve temporal resolution and accuracy.

Several efforts have been made to develop innovative approaches to detecting change using SAR data. The most common conventional SAR-based change detection approaches involve image rationing [2], image differencing [3], principal component analysis [4], multi-date image classification [5,6,7], and change vector analysis [8]. Similarly, several researchers have focused on model-based change detection by exploiting SAR time-series images. Yakoub Bazi et al. (2005) proposed an unsupervised approach based on a generalized Gaussian model to detect change using multi-temporal SAR images [9]. Wen Yang et al. (2016) employed Wishart mixture models for change detection in polarimetric SAR images. The proposed method was evaluated using datasets from RADARSAT-2 and ALOS PALSAR. Both qualitative and quantitative assessments demonstrated the superiority of the Wishart-mixture-model-based approach over conventional pixel-based techniques [9]. Jordi Inglada et al. (2007) utilized a new statistical similarity to perform multi-scale change analysis on multi-temporal SAR images [10].

Very limited efforts have been made to utilize KOMPSAT-5 images for change detection applications. Choi et al. (2022) proposed a scale-adaptive difference image (SADI) approach combined with morphological filtering to enhance change detection in KOMPSAT-5 SAR imagery. The method dynamically adjusts to different scales of change and refines results through noise suppression and shape continuity improvement. Experimental results demonstrate high accuracy in detecting diverse change targets, making it suitable for urban monitoring and environmental analysis [11]. Chae et al. (2022) developed a prototype program for automatic change detection by exploiting multi-temporal KOMPSAT-5 SAR imagery. The program integrates pre-processing, feature extraction, and change detection algorithms to automatically identify areas of significant change [2]. KOMPSAT-5 SAR data were also utilized to detect urban changes caused by earthquakes, focusing on structural damage and surface deformation. The proposed approach accurately identifies affected areas, highlighting KOMPSAT-5 data’s effectiveness for post-disaster urban monitoring [12].

In this research, a statistical homogenous pixel selection algorithm is being utilized along with a time-series coherence estimate to detect changes in the urban environment in KOMPSAT-5 time-series images. A statistical homogenous pixel selection algorithm is mainly being utilized for distributed scatterer target extraction for land deformation mapping [13]. Mainly, there are two types of homogeneous pixel selection approaches: non-parametric testing methods and parametric testing methods. Non-parametric testing methods primarily include the two-sample Kolmogorov–Smirnov (KS)/Anderson–Darling test [14] and Baumgartner–Weiss–Schindler (BWS) test [15]. The Kolmogorov–Smirnov (KS) and AD test algorithms offer notable advantages, including a well-defined sampling distribution for the test statistic and the ability to calculate the rejection region using an analytical formula. However, the KS and AD tests are less sensitive to differences in the tails of the sample distributions [16]. The SHP selection algorithm is mainly used in time-series interferometric SAR (InSAR) analysis for coherent phase detection over non-urban regions [17,18]. In this research, the SHP selection algorithm is proposed for change detection for the first time. Additionally, a comprehensive methodological framework is introduced for KOMPSAT-5-based change detection in urban areas.

This paper is organized as follows: Section 2 mainly focused on the description of the study site, SAR and ancillary dataset used in this research, and proposed methodological framework adopted for change detection using KOMPSAT-5 time-series images. In Section 3, obtained results are analyzed and discussed. In Section 4, study is concluded along with future directions.

## 2. Materials and Methods

### 2.1. Statistical Homogeneous Pixels Selection Algorithm Implementation

The statistical homogenous pixel selection utilizes the Anderson–Darling (AD) goodness-of-git test to select SHPs. Only pixels with the same class are tested by the AD test. The procedure of a statistical homogeneous pixels selection algorithm is schematically described as follows:

1. Compute the average of N temporal and spatial pixels to derive the amplitude image. The normality of $\bar{A}$ is then evaluated over a homogeneous region using the Anderson–Darling (AD) goodness-of-fit test.
2. Select a region presumed to be homogeneous to serve as a reference for analysis
3. Apply the Anderson–Darling (AD) goodness-of-fit test to $\bar{A}$ to determine whether the data conforms to a normal distribution
4. Define a significance level to classify pixels, accepting or rejecting them based on the results of the AD test.
5. Identify and output the resultant SHPs, which represent statistically stable pixels suitable for further analysis in time-series images.

### 2.2. Study Site

**Figure 1:**
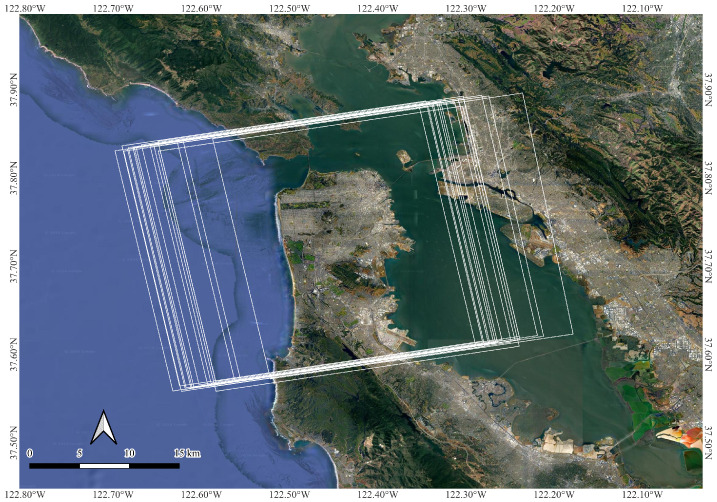
Location of study site along with KOMPSAT-5 time-series image footprints.

San Francisco, the fourth most populous city in California, United States, was selected as the study site for this research. The city’s varied topography, encompassing urban landscapes, hilly terrain, and coastal areas, provides a range of land cover types and structural features, making it an ideal environment for synthetic aperture radar (SAR)-based change detection studies. Figure 1 shows the geographical location of the study site and KOMPSAT-5 time-series image footprints covering the study site.

### 2.3. SAR and Ancillary Datasets Used

Time-series data from KOMPSAT-5, acquired in single-polarization (HH) mode, were obtained from the Korea Aerospace Research Institute (KARI) in single-look complex (SLC) format. Specifications of the KOPMSAT-5 satellite data are shown in Table A1. A total of 36 images covering the study site were collected from January 2021 to December 2023. The specifications of KOMPSAT-5 time-series acquisitions are listed in Table A2. The footprints of KOMPSAT-5 imagery over the study site are shown in Figure 1.

**Figure 2:**
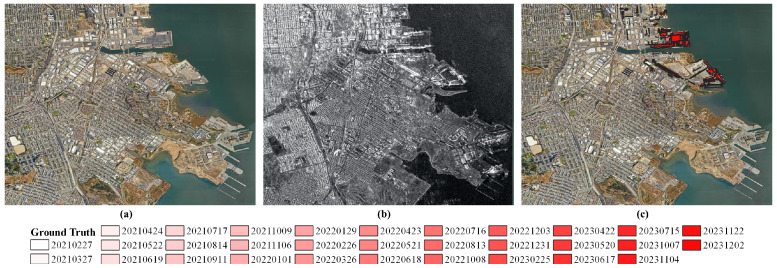
Dataset for change detection analysis: (**a**) optical footprint of the study site (source: Google Earth; image acquisition date: 4 October 2024), (**b**) KOMPSAT-5 SAR imagery of the study site, and (**c**) generated ground truth for accuracy assessment.

As can be seen from Figure 1, KOMPSAT-5 time-series images were acquired with unstable orbit with perpendicular baseline ranging from 7291 m to −1392 m. To validate change detection results, ground truth data were generated through visual inspection of time-series KOMPSAT-5 images in a sequential manner. Figure 2 shows the optical footprint, KOMPSAT-5 image, and generated ground truth of the study site.

### 2.4. Methodological Framework

The preprocessing of KOMPSAT-5 time-series images follows a systematic workflow to ensure data consistency and accuracy for change detection analysis. The process begins with image coregistration, aligning each time-series image to a common reference to correct for any spatial misalignments across the stack. The coregistration of images is implemented using a cross-correlation technique. As this method can be computationally slow for very large search windows, the process is typically divided into two main steps: coarse and fine coregistration. In the coarse coregistration step, offsets are approximated using satellite orbit and timing data as a reference and/or by identifying approximate common points in the reference and secondary images. These points are then refined through correlation matching using large search windows (128 × 128). The fine coregistration step follows, employing an automated correlation technique to achieve sub-pixel alignment accuracy. Once the coregistration offsets are determined, the coregistration polynomial model (CPM) is estimated, and interferometric resampling is performed to align the secondary images with the reference geometry [19].

This co-registered stack is then calibrated to $\beta_{o}$, converting raw SAR data to a calibrated backscatter coefficient representing surface reflectivity. The Lee Sigma temporal speckle filter, with a window size of 7 × 7 and a sigma value of 0.9, is applied to reduce inherent SAR noise, thereby enhancing image clarity and improving interoperability within the time-series stack. Radiometric calibration is performed to correct variations in radar reflectivity due to changing topography, ensuring that backscatter values accurately represent the surface properties across all images. Terrain correction is applied next to compensate for distortions introduced by topographic variations, allowing each pixel to define a consistent ground location. Additionally, local incidence angle normalization adjusts the variations in radar incidence angles due to terrain slope, further standardizing backscatter values. Contrast-limited adaptive histogram equalization (CLAHE) [20] stretch is then performed to enhance the image contrast, making subtle features more discernible; CLAHE stretch was applied to time-series acquisition with the same configuration to keep radiometric homogeneity intact. Finally, data normalization is applied to normalize the processed data between 0 and 1, ensuring uniform data distribution across the time-series images. Data normalization ensures the standardization of time-series data and enhances the selection of statistically homogeneous pixels. By normalizing the data, variations in intensity are more effectively captured relative to the overall value range, facilitating the distinction between homogeneous and heterogeneous regions.

**Figure 3:**
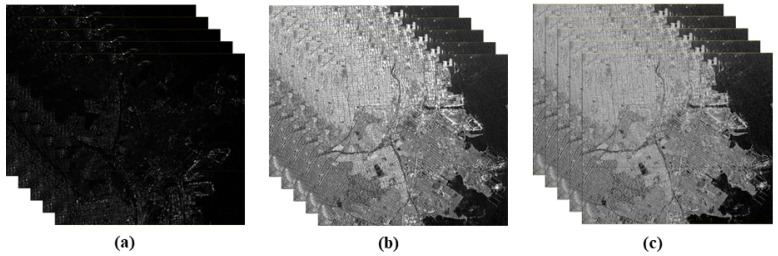
Preprocessing of KOMPSAT-5 time-series images: (**a**) KOMPSAT-5 time-series stack, (**b**) KOMPSAT-5 radiometric terrain-corrected time-series stack, (**c**) KOMPSAT-5 radiometric terrain-corrected normalized time-series stack.

These preprocessing steps are essential for producing a high-quality, consistent KOMPSAT-5 dataset, ready for detailed change detection analysis. The KOMPSAT-5 time-series stack before preprocessing and after radiometric terrain correction and normalization is shown in Figure 3. KOMPSAT-5 SAR data are also being utilized to detect urban changes caused by earthquakes, focusing on structural damage and surface deformation. The proposed approach accurately identifies affected areas, highlighting KOMPSAT-5 data’s effectiveness for post-disaster urban monitoring.

Similarly, the selection of statistical homogeneous pixels (SHPs) is performed to identify stable reference points, which are critical for maintaining consistency and accuracy in time-series analysis. This rigorous preprocessing pipeline ensures a high-quality, normalized dataset suitable for reliable change detection. However, the resultant SHPs often exhibit a salt-and-pepper effect due to their random distribution. To address this, a refined Lee despeckle filter with a 7 × 7 window size was applied, further enhancing image quality by reducing residual noise and producing a clean, coherent dataset.

**Figure 4:**
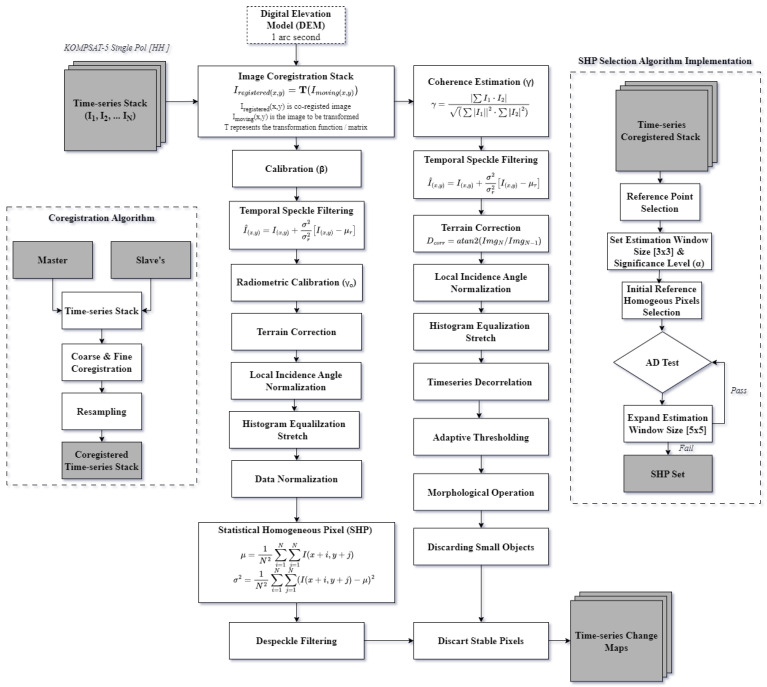
Detailed methodological framework adopted for change detection using KOMPSAT-5 images.

To detect the change in the time-series stack, coherence was estimated, using the co-registered stack to calculate coherence values, which serve as indicators of surface stability or change over time. Temporal speckle filtering is applied to reduce noise, enhancing the clarity of change patterns across the time-series KOMPSAT-5 images. Terrain correction adjusts for topographic distortions, ensuring that all detected changes correspond accurately to their geographic locations. Local incidence angle normalization and contrast-limited adaptive histogram equalization (CLAHE) stretch were performed to enhance local details and compensate for the difference in coherence due to the change in image acquisition geometry. Sequential time-series decorrelation estimation is performed next, analyzing the temporal evolution of coherence loss to identify potential changes. This step is followed by adaptive thresholding, which dynamically adjusts detection sensitivity to highlight changed pixels based on decorrelation patterns. Morphological operations, specifically opening and closing, are then applied to refine the detected regions by smoothing and connecting areas, improving shape accuracy and continuity. Small objects (≦20 pixels), which may represent noise or irrelevant features, are discarded to focus on significant changes. Additionally, pixels identified as stable through statistical homogeneous pixel analysis are excluded, ensuring that only true change areas are retained. The resultant change map highlights significant change within the study area, providing an accurate and noise-reduced output for further analysis. The detailed methodological framework adopted for this research is shown in Figure 4.

## 3. Results and Discussions

**Figure 5:**
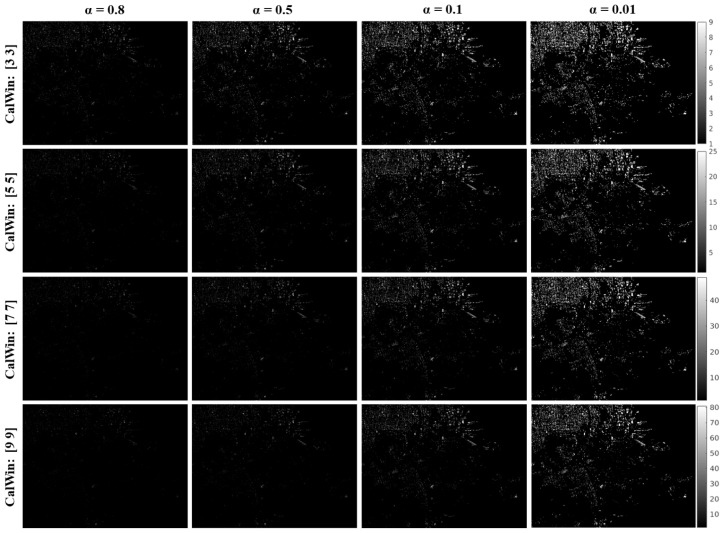
Experimental results to obtain appropriate statistical homogeneous pixels (SHPs).

By utilizing the Anderson–Darling goodness-of-fit test, statistical homogeneous pixels (SHPs) were selected. The key parameters for SHP selection are the significance level (α) and kernel size. To identify appropriate SHPs representing stable targets in KOMPSAT-5 images, the α value was varied from 0.01 to 0.8 in increments of 0.01, while the kernel size (CalWin) ranged from 3 × 3 to 15 × 15 pixels, increasing by 2. The resultant SHPs are shown in Figure 5. As can be observed in Figure 5, with a significance level of α = 0.8, kernel sizes of 3 × 3, 5 × 5, 7 × 7, and 9 × 9 failed to detect stable targets precisely. However, at α = 0.5, major urban segments were detected as stable targets. Similarly, at α = 0.1, both major and small urban segments were identified as stable targets. Furthermore, at α = 0.01 and a kernel size of 3 × 3, individual urban areas, along with larger and smaller urban segments, were effectively detected as stable targets.

**Figure 6:**
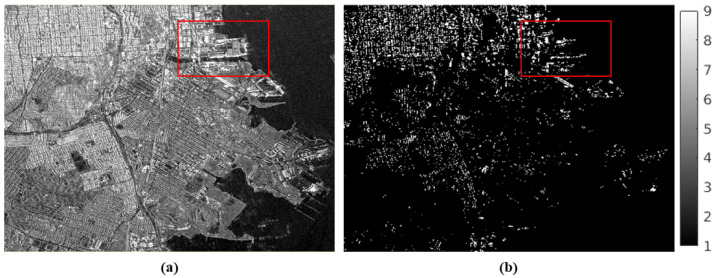
Statistical homogeneous pixels (SHPs) selection: (**a**) KOMPSAT-5 image, (**b**) resultant SHPs over urban segments. The San Francisco port area, highlighted within the red box, was selected for time-series change detection using the proposed technique.

By analyzing the resultant SHPs, those corresponding to α = 0.01 and a kernel size of 3 × 3 were selected as stable targets. The experimental results for the SHPs are shown in Figure 5. Figure 6b illustrates the selected SHPs over the urban segments. It is evident from the comparison between Figure 6a,b that most urban segments in the study site are relatively stable because major urban segments appeared as SHPs. However, the port area appears to be unstable; for this reason, change detection primarily focuses on the port area of San Francisco.

**Figure 7:**
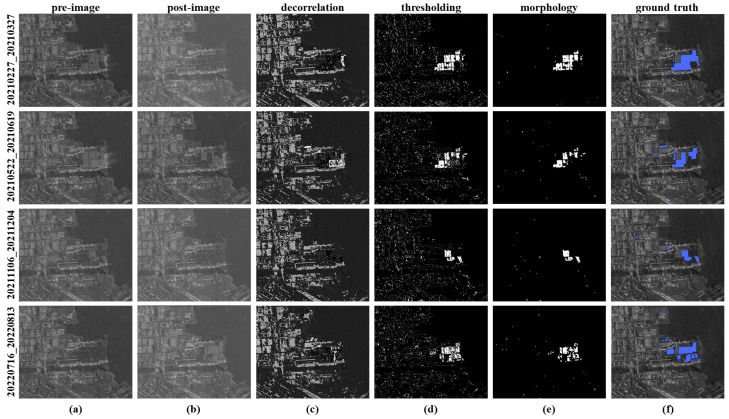
Change detection results by utilizing KOMPSAT-5 time-series images: (**a**) pre-image, (**b**) post-image, (**c**) de-correlation between pre- and post-image, (**d**) adaptive thresholding results, (**e**) detected changed area, (**f**) ground truth data.

Figure 7 presents the following: (a) the preprocessed pre-KOMPSAT-5 image, (b) the post-KOMPSAT-5 image utilized for change detection, (c) the correlation estimate between the pre- and post-images, (d) the binary image derived through adaptive thresholding, (e) the final change detection results highlighting the changed areas, and (f) the ground truth inventory data. As depicted in Figure 7, the changed areas between the pre- and post-images are represented as dark regions in the correlation estimate. This correlation estimate was subsequently refined through adaptive thresholding to delineate the changed areas.

The unstable InSAR orbit of KOMPSAT-5, with a perpendicular baseline variation between 7291 m and 1392 m, led to inconsistent projections of urban features across images. This resulted in significant misalignments within the co-registered time-series stack, which manifested as false changes in the adaptive thresholding process. Morphological opening and closing operations were employed to mitigate these false detections, effectively addressing most of the misalignments. Nevertheless, smaller false detections persisted, producing a salt-and-pepper noise effect. To resolve this, segments smaller than 20 pixels were eliminated after evaluating various segment size thresholds ranging from 5 to 30 pixels. While this method successfully suppressed the salt-and-pepper effect, it also inadvertently removed several minor changes within urban areas. To assess the reliability of the change detection results, an accuracy assessment was performed using the confusion matrix method. The overall accuracy (OA) of urban change detection was determined to be 92%.

A comparative analysis of the proposed technique with the method presented by Choi et al. (2022) [11] demonstrates that the proposed technique enhances change detection accuracy, increasing it from 90% to 92%. The improvement in accuracy is attributed to the capability of the statistical homogeneous pixel selection algorithm to accurately identify stable pixels, this prevents these pixels, which might otherwise be misclassified as false alarms, from being included in the change detection results. If the proposed technique is applied to SAR data acquired from satellites with stable orbits (e.g., TerraSAR-X, COSMO-SkyMed, RADARSAT), the overall accuracy is expected to improve. The stable orbital configurations of these satellites ensure consistent imaging geometry and reduced temporal and spatial decorrelation, enhancing the reliability of change detection results. A comparative performance evaluation of the proposed technique against the prototype method developed by Chae et al. (2022) [2] demonstrated that the proposed approach is much faster. While the method by Chae et al. (2022) requires approximately 40 min to generate a change map between two KOMPSAT-5 acquisitions, the proposed technique completes a time-series change map using 37 KOMPSAT-5 images in only 10 min. A comparison of the experimental results obtained using the proposed technique with those reported in the existing literature on KOMPSAT-5-based change detection is presented in Table A3.

## 4. Conclusions

This study presents a novel change detection methodology aimed at identifying urban changes using KOMPSAT-5 time-series data. The approach leverages the statistical homogeneous pixels (SHPs) selection method to extract stable targets and utilizes coherence history to quantify de-correlation caused by changes between the pre- and post-images. Misalignment artifacts introduced by the unstable InSAR orbit of KOMPSAT-5 were mitigated through the application of morphological opening and closing operations, along with the removal of small segments (≦20 pixels) to produce an accurate urban change map. The performance of the proposed method was rigorously evaluated using the confusion matrix technique, yielding an overall accuracy of 92%. The findings suggest that the accuracy of the proposed change detection approach could be further improved with stable InSAR orbit data. This methodology demonstrates significant potential for effective and reliable change detection in urban environments.

## Data Availability

Data are contained within the article.

## Abbreviations

The following abbreviations are used in this manuscript:

AD	Anderson–Darling
CLAHE	Contrast-limited adaptive histogram equalization
CPM	Coregistration polynomial model
DEM	Digital elevation model
KOMPSAT-5	Korea Multi-Purpose Satellite-5
OA	Overall accuracy
SHPs	Statistical homogenous pixels
SLC	Single-look complex
SNAP	Sentinel Application Platform
SRTM	Shuttle Radar Topographic Mission

## Appendix A

**Table A1:** Specifications of KOMPSAT-5 SAR satellite imagery utilized in this study.

Parameter	KOMPSAT-5
Band	X-band
Acquisition Mode	Enhanced Standard
Multi-Beam ID	ES-05
Orbit Direction	Ascending
Look Direction	Right
Mean Incidence Angle (deg)	30.27
Azimuth Pixel Spacing (m)	2.05
Ground Range Pixel Spacing (m)	1.94
Swath	30 km
Polarization	HH
Positional Accuracy	6.22 m CE90 absolute

**Table A2:** KOMPSAT-5 data used in this study with incidence angle = 31.57°, range x azimuth resolution (m) = 1.94 × 2.04, and mode of acquisition is Single Pol (HH).

Date of Acquisition (YYYYMMDD)
20210130
20210227
20210327
20210424
20210522
20210619
20210717
20210814
20210911
20211009
20211106
20211204
20220101
20220129
20220226
20220326
20220423
20220521
20220618
20220716
20220813
20221008
20221203
20221231
20230225
20230325
20230422
20230520
20230617
20230715
20231007
20231104
20231122
20231123
20231202
20231220
20231230

**Table A3:** Comparison of experimental results with existing literature on KOMPSAT-5-based change detection. The computer specifications used by [2] include an Intel^®^, Core^TM^ i7-10700 CPU @ 2.90 GHz with 32.0 GB of memory. The specifications of the computer used by [11] are not reported. For processing KOMPSAT-5 time-series images with the proposed techniques, an Intel^®^, Xeon^®^, Silver 4114 CPU @ 2.20 GHz with 256.0 GB of memory was utilized.

	Input Image Size	Processing Time	Overall Accuracy
Proposed Technique	2500 × 2500	2	92%
	5000 × 5000	4	92%
	10,000 × 10,000	10	92%
Chae et al. (2022) [2]	2500 × 2500	3	Not reported
	5000 × 5000	11	Not reported
	10,000 × 10,000	40	Not reported
Choi et al. (2022) [11]	2500 × 2500	Not reported	90%
	5000 × 5000	Not reported	90%
	10,000 × 10,000	Not reported	90%
